# Breakthroughs of CAR T-cell therapy in acute myeloid leukemia: updates from ASH 2024

**DOI:** 10.1186/s40164-025-00651-6

**Published:** 2025-04-11

**Authors:** Haixiao Zhang, Hong-Hu Zhu

**Affiliations:** 1 Department of Hematology, Beijing Chao-Yang Hospital, Capital Medical University, Beijing, 100020 China; 2 Chinese Institute for Medical Research, Capital Medical University, Beijing, 100069 China

## Abstract

While chimeric antigen receptor (CAR) T-cell therapy has revolutionized the treatment landscape for lymphoid malignancies, its greatest challenge remains in the treatment of acute myeloid leukemia (AML). Its success in AML has been limited by the ideal target antigen, myelosuppression, and immunosuppressive leukemia microenvironment. The 2024 ASH Meeting highlighted several cutting-edge advancements in AML-directed CAR T therapies, including clinical trials targeting CD33, CD123, CLL1, CD19, and IL1RAP, as well as novel engineering strategies such as dual-targeting CARs, inhibitory CAR designs, and genome-editing approaches to enhance safety and efficacy. Here, we summarize key findings from both clinical and preclinical studies, offering insights into the evolving landscape of CAR T-cell therapy for AML.

## To the editor

Refractory and relapsed (r/r) acute myeloid leukemia (AML) remains a therapeutic challenge with dismal prognosis, necessitating innovative approaches. The encouraging results of chimeric antigen receptor (CAR) T-cell therapy in other hematologic malignancies have spurred its investigation in AML. However, challenges such as antigen heterogeneity, challenging tumor microenvironment and off‑target toxicity complicate its clinical application. Despite these obstacles, ongoing efforts drive clinical and pre-clinical advancements, as highlighted in the 2024 ASH Meeting.

## Clinical studies

Early-phase trials targeting diverse antigens demonstrate variable efficacy and safety profiles (Table 1**)**. Tong et al. evaluated CD33 CAR T-cells in 12 post-transplant relapse patients, reporting a complete remission (CR) rate of 41.67% without cytokine releasing syndrome (CRS) greater than grade 2 or immune effector cell-associated neurotoxicity syndrome (ICANS) [1]. Swat et al. investigated CD123 CAR T-cells manufactured with Dasatinib (CD123-CAR.dasa T-cells) in six patients (5 AML, 1 ALL). Although CD123-CAR.dasa T-cells expanded effectively, they did not improve efficacy compared to CD123 CAR T-cells and induced grade ≥ 2 CRS in all 6 patients, underscoring toxicity concerns [2].

**Table 1:** Outcomes of clinical trials of CAR T-cells presented at ASH 2024

Targets	NCT number	Source	Dose (10e6/kg)	Patients (*n*)	CR (*n*)	Side effects		
						CRS (grade)	ICANS (grade)	Others
CD123	NCT04318678^*^	auto	3	3	NR	G 2, *n* = 3	NR	NR
			10	3	NR	G 4–5, *n* = 3		IEC-HS, *n* = 3
CD33	NR	auto	0.062–0.615	12	5	G 1, *n* = 9; G 2, *n* = 1	*n* = 0	liver function damage, *n* = 4; sepsis, *n* = 2
CLL1(CD371)	NCT06017258	auto	0.3	2	2	G 3–4, *n* = 1; G 1–2, *n* = 1	G 3, *n* = 1	DLT, *n* = 2
		auto	0.03	3	1	G 3–4, *n* = 1; G 1–2, *n* = 2		DLT, *n* = 1
	ChiCTR2000041054^†^	auto	NR	20 w/ EMDs	13	NR	NR	NR
				27 w/o EMDs	22			
	NR^‡^	healthy donor	3	3	2	G 3, *n* = 2	G1, *n* = 1	NR
CD19	NCT03896854^§^	auto	5–20	10	6	G 1∼2, *n* = 8;G 3, *n* = 1	NR	neutropenia or thrombocytopenia: G3, *n* = 3; G4, *n* = 7; G 1 liver dysfunction, *n* = 1; hypertension, *n* = 1
IL-1RAP	NCT06281847	auto	0.1, 0.5, 1, 5, 10	NR	NR	NR	NR	NR

* Six patients including 5 AML and 1 ALL, received CD123-CAR T cells generated in the presence of Dasatinib; † The aim is to compare the efficacy and safety between patients with EMDs and without EMDs; ‡ ThisCART371, containing a CLL1-targeted CAR and a KDEL-tagged anti-CD3 single chain antibody which prevents TCRαβ/CD3 from being secreted from the endoplasmic reticulum; § The aim to assess the safety and efficacy of CD19 CAR T-cell therapy in CD19-positive relapsed t(8;21) AML

NR, not recording; G, grade; w/, with; DLT, dose-limiting toxicity; IEC-HS, immune effector cell-associated HLH-like syndrome; w/o without; EMDs, extramedullary diseases;

Zhao et al. reported several results of CLL1 CAR T-cell in r/r AML. They enrolled 47 r/r AML patients received CLL1 CAR T-cell, including 20 (42.6%) patients with extramedullary infiltration. Notably, patients with or without extramedullary infiltration showed comparable over survival (OS), leukemia-free survival (LFS), and incidence rates of complications [3]. In addition, modified CLL1-CARs incorporating KDEL-tagged anti-CD3 (ThisCART371) was used in three patients and two of them achieved CR; unfortunately, all died [4]. Infection after CAR T-cell therapy is also a serious concern and Zhao et al. also shared their experience in infections after CLL1 CAR T-cell in 51 patients, showing a 28-day cumulative rate of bacterial, fungal and viral infections at 56.9% (95% CI 50.4%-61.3%), 15.6% (95% CI 11.7%-19.1%) and 11.7% (95% CI 9.3%-14.8%), respectively [5].

Moreover, a phase I trial of the novel CD371-SAVVYz-IL18 CAR T-cell, engineered with a modified CD28 costimulatory domain to limit T cell exhaustion and with constitutive IL-18 secretion to boost cytotoxicity, demonstrated minimal residual disease (MRD)-negative CR in three of five patients, though grade 2–3 CRS and grade 3 ICANS were observed [6].

CD19 is frequently expressed on t(8;21) AML and can serve as a therapeutic target. A trial of CD19 CAR T-cell in ten t(8;21) AML patients showed a high safety profile, without severe non-hematological toxicities, and remarkable efficacy, with a 100% response rate, including 60% attaining MRD-negative CR. The 12-month OS and LFS was 45.0% and 46.7%, respectively [7]. Ongoing trials like RESOLVE-AML 001 (IL1RAP-targeted CCTx-001) may expand targetable epitopes.

## Preclinical innovations

**Fig. 1:**
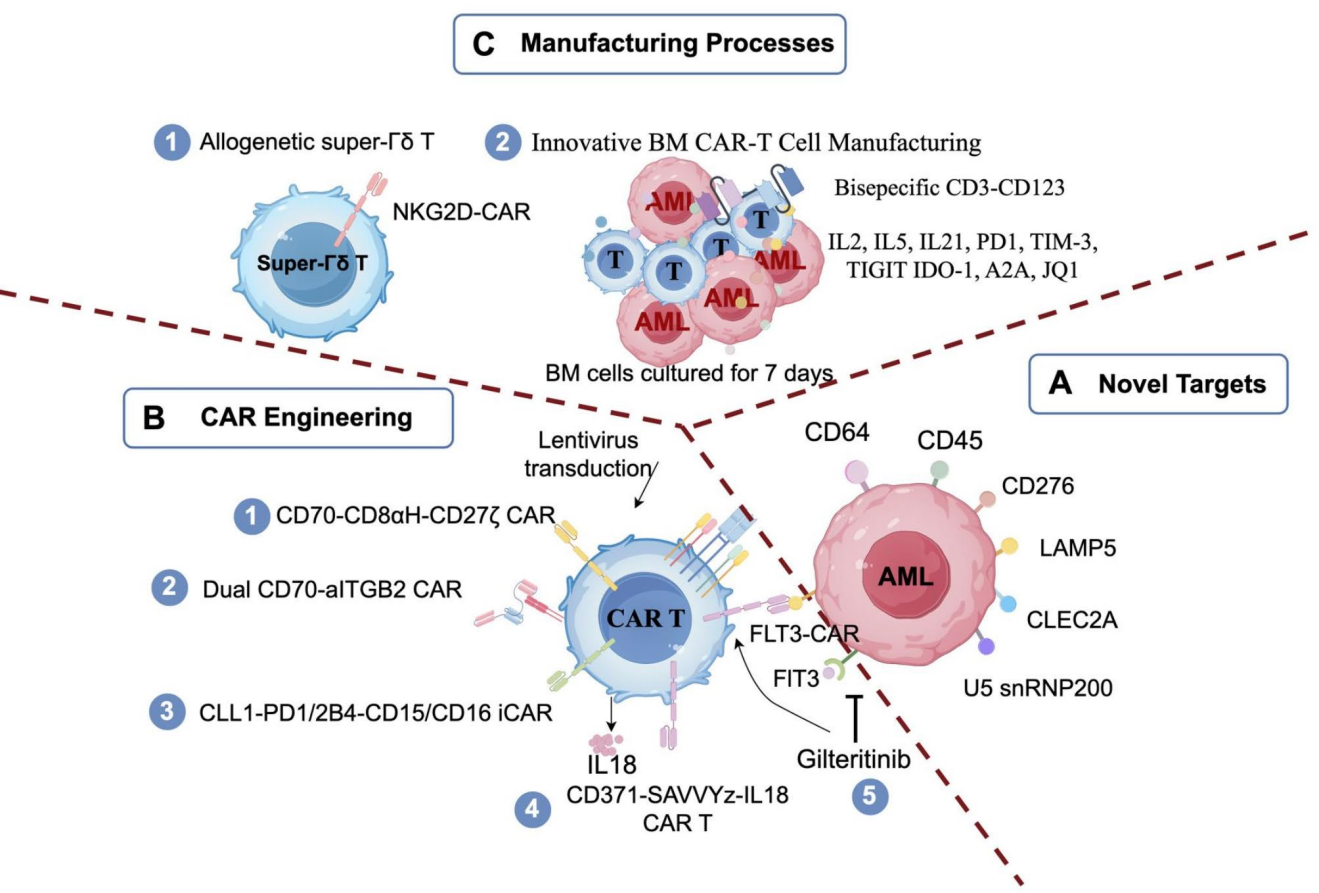
Pre-clinical innovations in CAR T-cells for acute myeloid leukemia. **A** Novel Targets, summarizing newly explored targets for CAR T-cell in AML. **B** CAR Engineering, detailing structural modifications, optimizations, and combination strategies for CAR T cells in AML. **C** Manufacturing Processes, highlighting advancements in CAR T-cell production

At this meeting, the preclinical studies primarily focused on novel targets, CAR engineering and CAR T manufacturing processes (Fig. 1). Promising AML-specific targets under investigation include U5 snRNP200, CLEC2A, CD276, LAMP5, CD64 and CD45. Notably, CD64-directed CAR T works well for Ven/Aza resistant, monocytic AML [8].

Innovative advances in CAR engineering have focused on improving safety, efficacy, and applicability for AML treatment. Pioneering innovations include inhibitory CAR architectures designed to mitigate on-target/off-tumor toxicity (exemplified by CD16-CLL1 CAR-mediated neutrophil preservation) and machine learning-optimized constructs that maximize signaling fidelity [9, 10]. The therapeutic landscape is further being reshaped by two paradigm-shifting approaches: (i) the development of universal CAR-T platforms through multiplex genome editing, and (ii) the integration of bispecific targeting modalities with small molecule adjuvants to combat antigen escape and improve persistence (see Fig. 1 for detailed mechanisms).

On the manufacturing front, Bejarano Garcia et al. introduced an “Immunocoaching T Cells” approach using cryopreserved AML samples, bispecific CD3-CD123 antibodies, cytokines, and checkpoint inhibitors to enhance CAR T-cell antitumor activity and persistence [11]. Furthermore, the development of a Super γδ T-cell via site specific integration of an NKG2D based CAR using PrecisionGENE technology represents another exciting advancement [12].

In conclusion, although CAR T-cell therapy in AML has not yet replicated the success seen in B-cell malignancies, recent clinical and preclinical studies are laying the groundwork for overcoming existing challenges. Strategic integration of genome editing, refined CAR engineering and innovative manufacturing techniques holds significant promise for developing safer and more effective therapies for r/r AML.

## Data Availability

No datasets were generated or analysed during the current study.
